# Engineering of a Genetically Encodable Fluorescent Voltage Sensor Exploiting Fast Ci-VSP Voltage-Sensing Movements

**DOI:** 10.1371/journal.pone.0002514

**Published:** 2008-06-25

**Authors:** Alicia Lundby, Hiroki Mutoh, Dimitar Dimitrov, Walther Akemann, Thomas Knöpfel

**Affiliations:** 1 Laboratory for Neuronal Circuit Dynamics, RIKEN Brain Science Institute, Wako-City, Saitama, Japan; 2 The Danish National Research Foundation Centre for Cardiac Arrhythmia, University of Copenhagen, Copenhagen, Denmark; University of California Berkeley, United States of America

## Abstract

Ci-VSP contains a voltage-sensing domain (VSD) homologous to that of voltage-gated potassium channels. Using charge displacement (‘gating’ current) measurements we show that voltage-sensing movements of this VSD can occur within 1 ms in mammalian membranes. Our analysis lead to development of a genetically encodable fluorescent protein voltage sensor (VSFP) in which the fast, voltage-dependent conformational changes of the Ci-VSP voltage sensor are transduced to similarly fast fluorescence read-outs.

## Introduction

**Figure 1:**
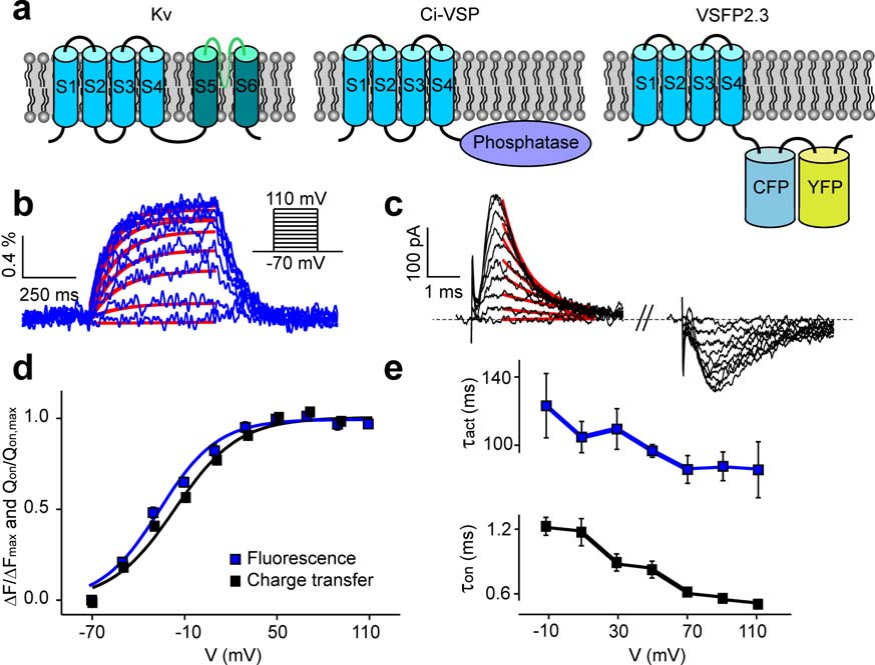
Fast voltage-dependent VSD movements and slow fluorescence signals in VSFP2.3. (a) Membrane topology of single Kv channel subunit, Ci-VSP and VSFP2.3. VSDs are shown in blue. (b) Change in yellow fluorescence induced by depolarizing voltage steps recorded from a PC12 cell stably expressing VSFP2.3. Red traces are single exponential fits. (c) On- and off ‘gating’ currents induced by the same voltage steps. The On-‘gating’ decay is fitted by single exponential functions (red traces). (d) Fluorescence-voltage (F-V) (n = 11, blue) and charge-voltage (Q-V) relations (n = 10, black) of VSFP2.3. (e) Voltage-dependency of time-constants for VSFP2.3 fluorescence activation (blue) and the decay of on-‘gating’ currents (black). Note broken time scale.

Cells use voltage sensor containing proteins to control the membrane potential and for signaling processes. Among these proteins are the extensively studied voltage-gated potassium channels (Kv channels), which are constituted by four homologous subunits each with transmembrane segments S1–S4 forming a voltage-sensing domain (VSD) and S5–S6 contributing to the pore structure (Figure 1a left) [1], [2]. Recently, a homolog to the VSD of Kv channels was discovered to be coupled to a phosphatase in the ascidian *Ciona intestinalis* (*Ciona intestinalis* voltage-sensor containing phosphatase; Ci-VSP) (Figure 1a middle) [3], and unlike Kv channel subunits Ci-VSP can exist in the membrane as a monomer [4]. The self-containing properties of the Ci-VSP voltage sensor makes it particularly suitable for the study of voltage-sensing mechanisms [5] and has enabled successful engineering of a protein for optical measurement of membrane potential (voltage-sensitive fluorescent protein; VSFP) [6]. Genetically encodable fluorescent voltage probes hold great promise in neuroscience, where methods that allow recordings of electrical activity from multiple identified neurons simultaneously are needed [7], [8]. The first VSFP based on the Ci-VSP voltage sensor, named VSFP2.1, was generated by fusing the VSD of Ci-VSP to a pair of cyan- and yellow-emitting proteins (cyan/yellow fluorescent protein; CFP/YFP) and introducing a R217Q mutation in S4 to shift the activation curve of the sensor into the physiological range of neuronal membrane potential [6]. Removal of five amino acids originating from engineered restriction sites in VSFP2.1 resulted in VSFP2.3, and both versions of the sensor exhibit excellent membrane targeting in PC12 cells (Figure 1a right, Figure S1). The main obstacle of these VSFP variants is that their fluorescence read-out is slower than required for measurement of fast electrical signals in neurons.

## Results and Discussion

**Figure 2:**
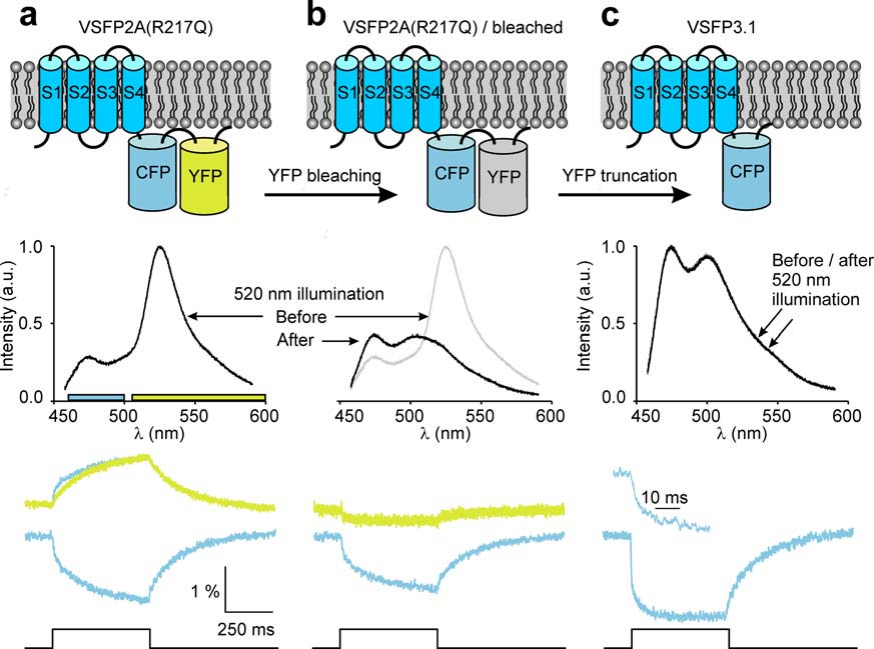
Development of a fast reporting VSFP. The membrane topology of VSFP2A(R217Q) (a), VSFP2A(R217Q) after photobleaching YFP (b) and VSFP3.1 (c) are shown in the top panel. Underneath are emission spectra recorded from each construct using 440 nm excitation light. The lower panel shows the fluorescence signals recorded in the yellow and cyan channels. For VSFP2A(R217Q) a scaled mirror-image of the cyan signal is shown aligned with the yellow signal; note the fast CFP component. For VSFP3.1 the onset of the fluorescence signal is shown on an expanded time scale; note the dramatically faster response of VSFP3.1.

**Figure 3:**
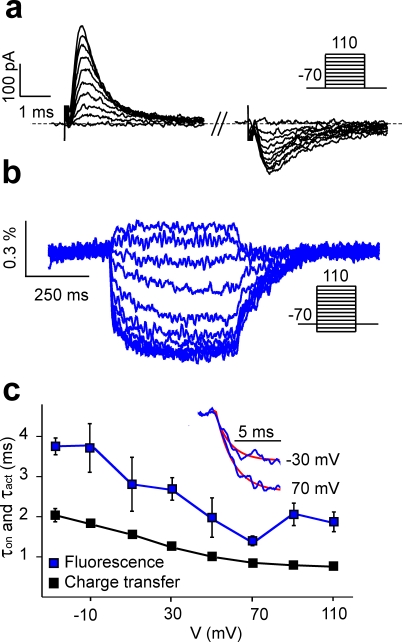
Characterization of VSFP3.1 ‘gating’ currents and fluorescence response. Representative ‘gating’ currents (a) and fluorescence responses (b) of VSFP3.1 elicited from single cells upon a family of voltage steps. (c) Voltage-dependency of the fast component of the fluorescence activation time-constant (blue) and the time-constant for the decay of on-‘gating’ currents (black). The inset shows single exponential fits to the fast fluorescence time-constant component from averaged fluorescence traces (n = 8) obtained with voltage steps to −30 mV and +70 mV.

With the goal to improve the VSFP response kinetics we set out to investigate the molecular activation mechanism of VSFP2.3. The conformational transitions of the protein upon a voltage change are initiated by displacement of charged amino acids of the VSD, which gives rise to a transient current analogous to the gating currents known from ion channels [9]. Measurement of such ‘gating’ currents of Ci-VSP in *Xenopus* oocytes [3] suggests that VSD rearrangements in Ci-VSP are slow compared to VSD movements in most ion channels [10]. To address if the slow fluorescence response of VSFP2.3 is due to intrinsically slow operations of its VSD, we measured fluorescence signals along with ‘gating’ currents in a PC12 cell-line stably expressing VSFP2.3 (Figure S2, Supplementary Methods). The voltage-dependency of the fluorescence read-out closely resembles the activation curve for charge displacement (Figure 1b–d) indicating that the fluorescence signal reports the voltage-dependent conformation of the VSD. However, we found the ‘gating’ charge movement to be two orders of magnitude faster than the fluorescence response (∼1 ms versus ∼100 ms) (Figure 1e), and more than ten times faster than the reported Ci-VSP ‘gating’ currents recorded from *Xenopus* oocytes (∼30 ms) [3]. The surprising rapidity of the ‘gating’ currents might be related to the R217Q mutation in S4. However, measuring charge displacements from VSFP2.3 Q217R revealed similarly fast ‘gating’ currents (1.9±0.1 ms at 70 mV, n = 10, data not shown). It thus appears that VSD movements in VSFP2.3 are intrinsically fast, and that the slow fluorescence report instead reflects weak coupling between the VSD and the fluorescent reporter proteins (FPs). We made two important observations concerning the effect of VSD-FP coupling: First, the ratio of CFP to YFP emission of VSFP2.1 increased when the VSD-FP linker was shortened [6]. Second, VSFP2.1 in its short linker version (VSFP2A R217Q, Figure S1) clearly exhibited a fast component in CFP response, which is not present in the YFP signal (time-constants CFP: 7±2 ms and 180±15 ms, YFP: 171±15 ms, n = 8) (Figure 2a). These observations led us to hypothesize that stronger VSD-FP coupling, as presumably achieved by linker shortening, may give rise to a faster depolarization response in CFP independent of the CFP-YFP energy transfer mechanism (fluorescence resonance energy transfer; FRET). To test this hypothesis we photo-bleached the YFP chromophore subsequently to the measurements illustrated in Figure 2a. Single cell spectro-fluometry confirmed disappearance of the 530 nm YFP emission band and increase in CFP emission at 470 nm due to acceptor bleaching and donor de-quenching, respectively (Figure 2b). Importantly, the YFP response was completely abolished by photo-bleaching (the YFP channel even showed a small reversed signal caused by crosstalk from the CFP detection channel), whereas a significant CFP response remained (time constant of fast component 5±1 ms; n = 8). This experiment provides direct evidence that the fast-component CFP response does not require presence of a FRET acceptor. To test whether the presence of YFP is required for structural reasons or, in contrast, removal of the YFP domain would favor the fast intrinsic CFP response, we truncated the YFP domain and investigated the response properties of the construct obtained (VSFP3.1, Figure S1). VSFP3.1-transfected cells exhibited the well known emission spectrum of CFP, which was unaffected by the YFP bleaching protocol (Figure 2c). Most notably, VSFP3.1 responded to depolarizations with large response amplitudes and a very fast initial transient (activation time-constant 1.3±0.1 ms at 70 mV, n = 7). Linker shortening and truncation of YFP thus greatly improved the response time. Measurement of charge displacements in VSFP3.1 revealed similar voltage-dependency as found for VSFP2.3, as did measurements of the voltage-dependence of the fluorescence read-out (Figure 3a–b, Figure S3). The fluorescence output of VSFP3.1 exhibits a dominant time-constant that closely tracks the activation time-course of the charge movement (Figure 3c), and a less prominent slower component resembling the FRET component in the previous VSFP versions.

In conclusion, we find that the voltage sensor of Ci-VSP exhibits very fast ‘gating’ currents in mammalian cells, which underscores the notion that this VSD is functionally similar to that of Kv channels [5]. The unusual large difference in the kinetics observed in mammalian membranes as compared to *Xenopus* oocytes [11] may result from an expectedly strong dependence on the lipid environment of an isolated VSD [12]. Reverse engineering of the previously reported VSFP2.1 type of sensors revealed that their slow fluorescence reports are caused by inappropriate coupling between the VSD and the CFP/YFP domain. We accordingly re-engineered VSFP2.3 resulting in VSFP3.1, which displays fluorescence activation kinetics closely resembling the fast ‘gating’ currents. To our knowledge VSFP3.1 is the fastest FP voltage sensor that is functional in mammalian PC12 cells reported to date, exhibiting an activation time constant matching that of fast neuronal electrical signals. Future work will be aimed at a confirmation of the function and kinetic properties of VSFP3.1 in differentiated neurons, an important step towards realization of an optical sensor of neuronal circuit activity.

## Materials and Methods

### Cell and Molecular Biology

Constructs were prepared by modifying the previously published VSFP2A and VSFP2.1 plasmids^6^. The VSFP2.3 construct was generated by removal of the amino acids constituting the NotI (CGR) and BamHI (GS) restriction sites in VSFP2.1 using overlap-extension PCR. The R217Q mutation was introduced in VSFP2A by site-directed mutagenesis. VSFP3.1 was generated by substituting the FRET pair in VSFP2A(R217Q) with a single Cerulean using NotI and HindIII restriction sites. All constructs were verified by DNA sequencing.

PC12 cells (ATCC) were grown in high-glucose DMEM (Gibco-Invitrogen) supplemented with 5% fetal calf serum and 10% horse serum on poly-D-lysine coated coverslips. Tranfections were done one day after plating using Lipofectamine 2000 (Invitrogen) reagent according to the manufacturer's instructions. Recordings from transiently expressing PC12 cells were performed 48–72 h post transfection. PC12 cell-line stably expressing VSFP2.3 was established after 1 mg/ml G418 (Calbiochem) selection and clone expansion from limited dilutions at 0.1 cell/well in 96-well plates. Fluorescence signals and gating currents were recorded from either non-transfected PC12 cells, a PC12 cell-line stably expressing VSFP2.3 or from transiently transfected PC12 cells.

### Electrophysiology

A coverslip with PC12 cells was placed in a recording chamber mounted on the stage of an inverted microscope (Eclipse TE-2000, Nikon), and voltage-clamp recordings in the whole-cell configuration were performed using an Axopatch 200B amplifier (Axon Instruments). Clampex software (Axon Instruments) was used for data acquisition and for synchronization of voltage command pulses and fluorescence excitation. Borosilicate glass electrodes of a resistance of 3–5 MΩ were pulled on a two-stage vertical puller (PP-830, Narishige) and painted with Sylgard in two steps. Recordings were performed in a perfused chamber, and the bath temperature was kept at 25°C by a temperature controller. For each cell 3–7 current traces and 2–4 fluorescence traces were recorded and averaged. Recording solutions contained in mM (140 NMDG, 10 HEPES, 1 MgCl_2_, 1.8 CaCl_2_, 10 dextrose, pH 7.4 using HCl) for the pipette and (140 NMDG, 10 HEPES, 5 EGTA, 1 MgCl_2_, pH 7.2 using HCl) for the bath, where NMDG is N-methyl-D-glucamine. Fluorescence traces were elicited from a holding potential of –70 mV by a sequence of 20 mV activation steps lasting 500 ms to a final potential of 110 mV with 10 s interpulse intervals. ‘Gating’ currents were recorded by 20 ms potential steps in the range from –90 mV to +110 mV in 20 mV steps with a 5 s interpulse interval from a holding potential of –70 mV. Data was acquired at 50 kHz and filtered by a 5 kHz low pass Bessel filter.

### Fluorescence measurements

Fluorescence was induced by light (440 nm) from a computer controlled monochromator (Polychrome IV, T.I.L.L. Photonics) through a 50× oil immersion objective. Fluorescence emission was collected through the objective and directed via a first dichroic mirror (DCLP 445 nm) through a beam splitter (DCLP505) and an emission filter (D480/40) onto two photodiodes (Viewfinder, T.I.L.L. Photonics). Photodiode signals were digitized along with the electrophysiological signals using Axon hard- and software as described above.

For spectral measurements a fluorescence spectrometer (Fluorolog, HORIBA) was coupled to the inverted microscope by an optical fiber. Excitation (440 nm) and photo-bleaching (520 nm, 4 min exposure time) light were provided by a monochromator. For the data presented in Fig. 2 electrophysiology, fluorescence and spectral measurements were performed as a combined set of experiments on each cell tested.

### Data analysis

Fluorescence signals and gating currents were analyzed using Clampfit 9.2 (Axon Instruments), OriginPro 7 (OriginLab) and Excel (Microsoft) software.

### Fluorescence analysis

Photobleaching was corrected by subtraction of a bleaching curve (fluorescence trace at constant voltage). Fluorescence transients elicited by voltage steps were fit with single- and double exponential functions depending on what gave the best fit. The amplitude of fluorescence transients was measured at end of the test pulse and expressed as percentage of baseline fluorescence.

### ‘Gating’ current analysis

‘Gating’ currents were extracted from recorded current traces by subtraction of linear leak current and linear capacitive transients. Leak current was subtracted by measuring the mean current amplitude in the last 2 ms of the hyperpolarizing voltage step and subtracting a correspondingly scaled Ohmic current from each recorded current trace. Similarly, the capacitive transient needed to charge the cell membrane was estimated by a single-exponential fit to the current recorded at the hyperpolarizing voltage step, and a linear voltage dependent exponential was subtracted from each current trace recorded. The ‘gating’ current charge displacement was calculated as the time integral of the on- ‘gating’ current. Steady-state relations recorded from single cells were normalized and fit with two-state Boltzmann distributions. Values for half maximal activation and slope factors were calculated as mean values obtained from the ensemble of cells. All data points are represented by mean values and error bars indicate standard error of the mean.

For Figure 1d the Boltzmann fit parameters for F-V are V_1/2_ = −23.4±2.1 mV, slope factor a = 19.3±1.2 and for Q-V, V_1/2_ = −17.4±1.5 mV, slope factor a = 20.4±0.9. For the time constants shown in Figure 1e the values obtained at 70 mV are 85.8±8.1 ms for the fluorescence signal and 0.84±0.08 ms for the on-‘gating’ currents.

## Supporting Information

Figure S1 Sequences of VSFP constructs and expression in PC12 cells. (a) Amino acid sequences for VSFP constructs. The grey box indicates the putative VSD from Ci-VSP, and the blue and yellow boxes refer to cyan (CFP) and yellow (YFP) fluorescent proteins respectively. Amino acids are specified for regions containing modifications between different VSFP constructs. Unmodified regions are represented by amino acid numbering in brackets referring to the sequences of Ci-VSP VSD from Ci-VSP (NP_001028998), CFP from Cerulean A206K (CAP04994) and YFP from Citrine (AAV97899). R217Q mutation is shown in red. (b) Transmission (top panel) and fluorescence (lower panel) images of PC12 cells expressing VSFP2a(R217Q), VSFP2.3 and VSFP3.1. Notice the membrane targeting of VSFP constructs. Scale bar is 15 µm.
(6.81 MB TIF)

Figure S2 ‘Gating’ current measurements from non-transfected PC12 cells (controls) and PC12 cells stably expressing VSFP2.3. (a) Top: uncorrected current traces elicited from non-transfected PC12 cells by voltage steps ranging from −90 mV to +110 mV from a holding potential of −70 mV. Bottom: remaining currents after subtraction of linear leak current and capacitive transient. (b) Same experimental procedure using PC12 cells stably expressing VSFP2.3. The top trace shows uncorrected current traces as recorded. The bottom trace shows the remaining currents (‘gating’ currents) after subtraction of linear leak current and capacitive transient.
(2.42 MB TIF)

Figure S3 Voltage-dependency of VSFP3.1 fluorescence response and on-‘gating’ currents. (a) F-V relation for VSFP3.1 (n = 7). The change in cyan fluorescence elicited upon voltage steps between −110 mV and + 110 mV was normalized and fit to two-state Boltzmann distributions with mean values (V1/2 = −51.2±2.3 mV, a = 20.8±3.2). (b) Q-V relation for VSFP3.1 on-‘gating’ (n = 7). Currents were evoked by voltage steps between −70 mV and +110 mV, and the charge transfer was calculated and normalized. The data was fit to Boltzmann distributions with mean values (V1/2 = −18.3±1.5 mV, a = 19.1±1.2).
(0.76 MB TIF)
