# Supplementary figures and images for: Engineering of a Genetically Encodable Fluorescent Voltage Sensor Exploiting Fast Ci-VSP Voltage-Sensing Movements
Source: PLoS One. 2008 Jun 25;3(6):e2514. doi: 10.1371/journal.pone.0002514 (PMC2429971; doi:10.1371/journal.pone.0002514)

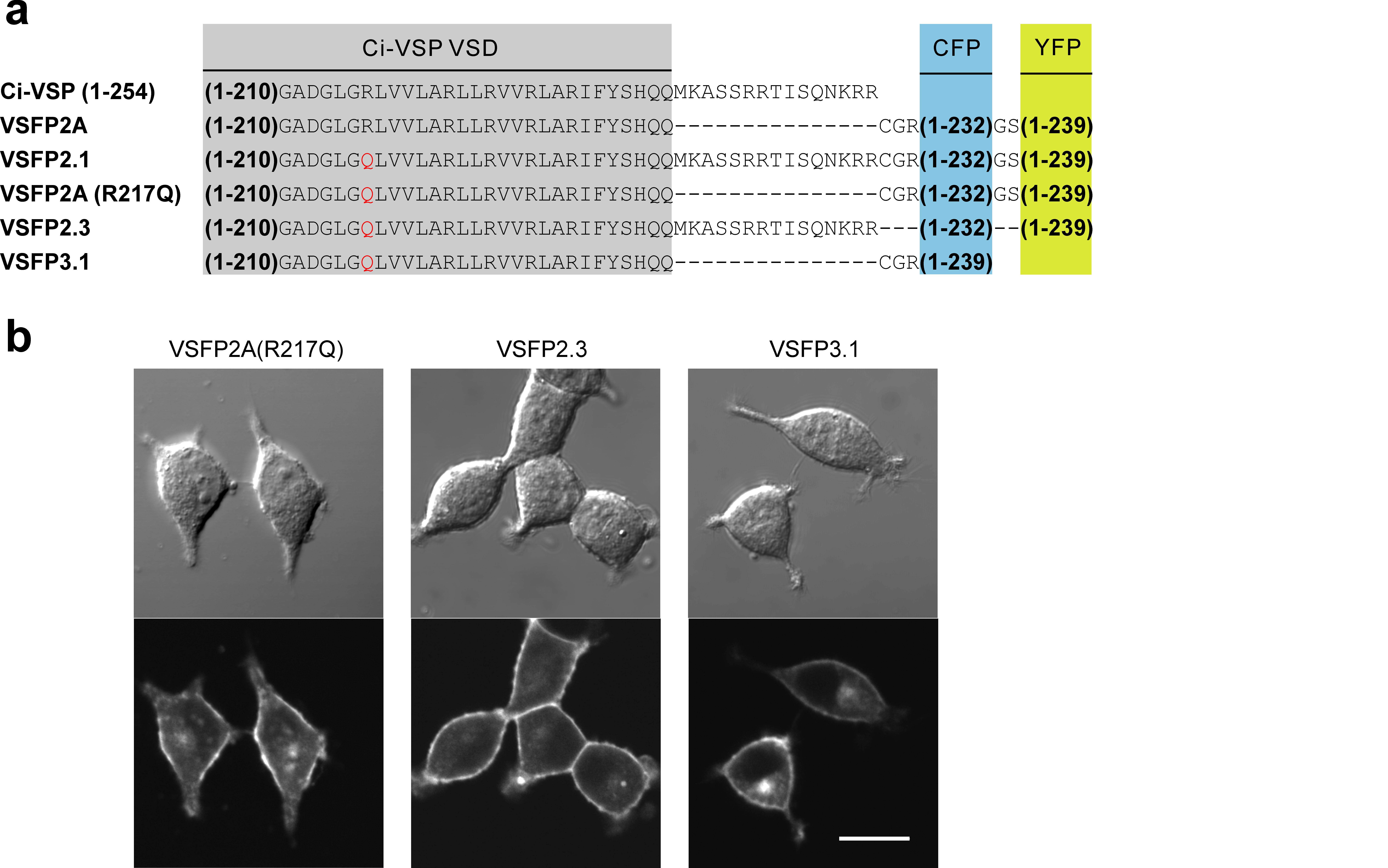

Supplement: Figure S1 — Sequences of VSFP constructs and expression in PC12 cells. (a) Amino acid sequences for VSFP constructs. The grey box indicates the putative VSD from Ci-VSP, and the blue and yellow boxes refer to cyan (CFP) and yellow (YFP) fluorescent proteins respectively. Amino acids are specified for regions containing modifications between different VSFP constructs. Unmodified regions are represented by amino acid numbering in brackets referring to the sequences of Ci-VSP VSD from Ci-VSP (NP_001028998), CFP from Cerulean A206K (CAP04994) and YFP from Citrine (AAV97899). R217Q mutation is shown in red. (b) Transmission (top panel) and fluorescence (lower panel) images of PC12 cells expressing VSFP2a(R217Q), VSFP2.3 and VSFP3.1. Notice the membrane targeting of VSFP constructs. Scale bar is 15 µm. (6.81 MB TIF) [file pone.0002514.s001.tif]

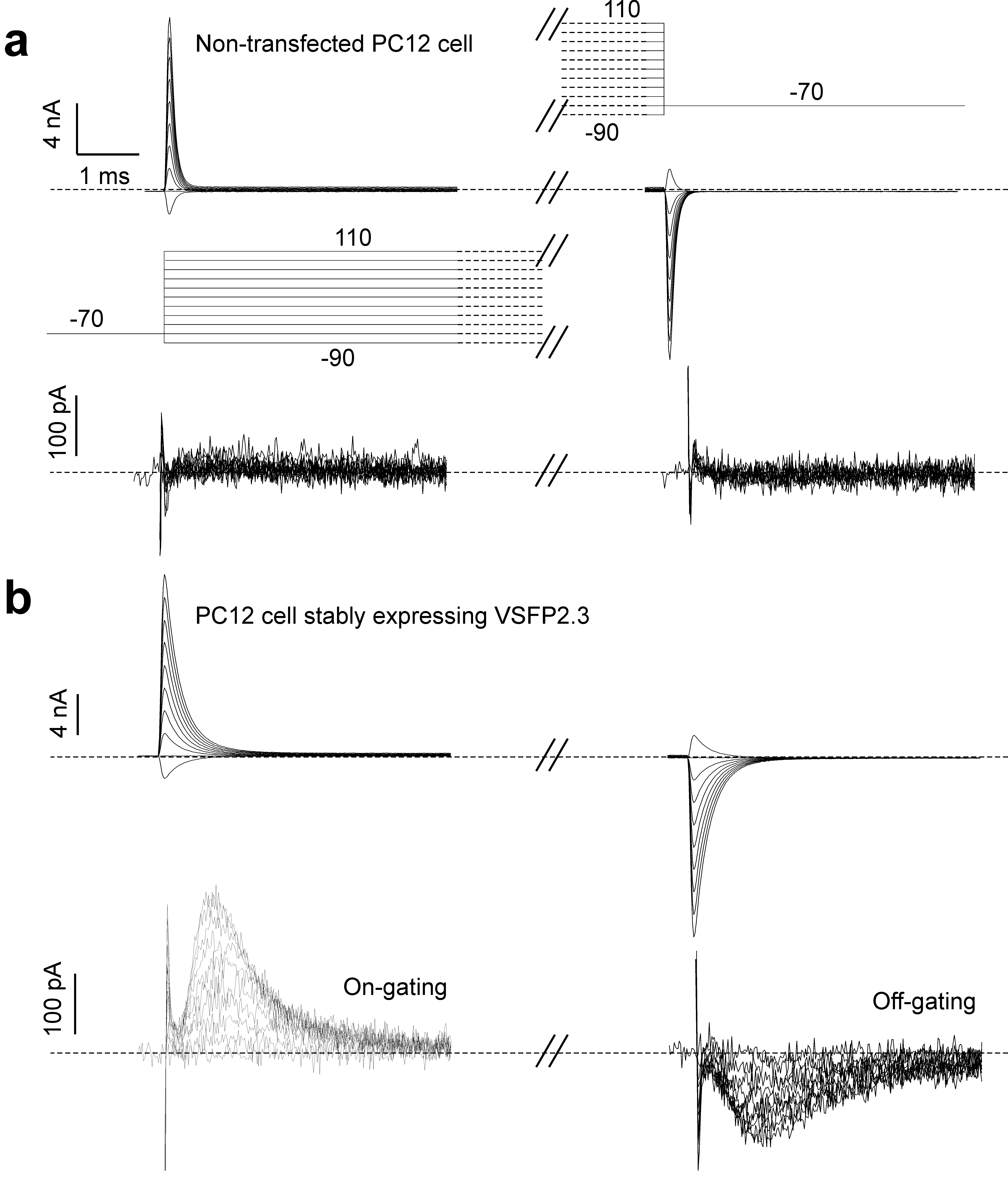

Supplement: Figure S2 — ‘Gating’ current measurements from non-transfected PC12 cells (controls) and PC12 cells stably expressing VSFP2.3. (a) Top: uncorrected current traces elicited from non-transfected PC12 cells by voltage steps ranging from −90 mV to +110 mV from a holding potential of −70 mV. Bottom: remaining currents after subtraction of linear leak current and capacitive transient. (b) Same experimental procedure using PC12 cells stably expressing VSFP2.3. The top trace shows uncorrected current traces as recorded. The bottom trace shows the remaining currents (‘gating’ currents) after subtraction of linear leak current and capacitive transient. (2.42 MB TIF) [file pone.0002514.s002.tif]

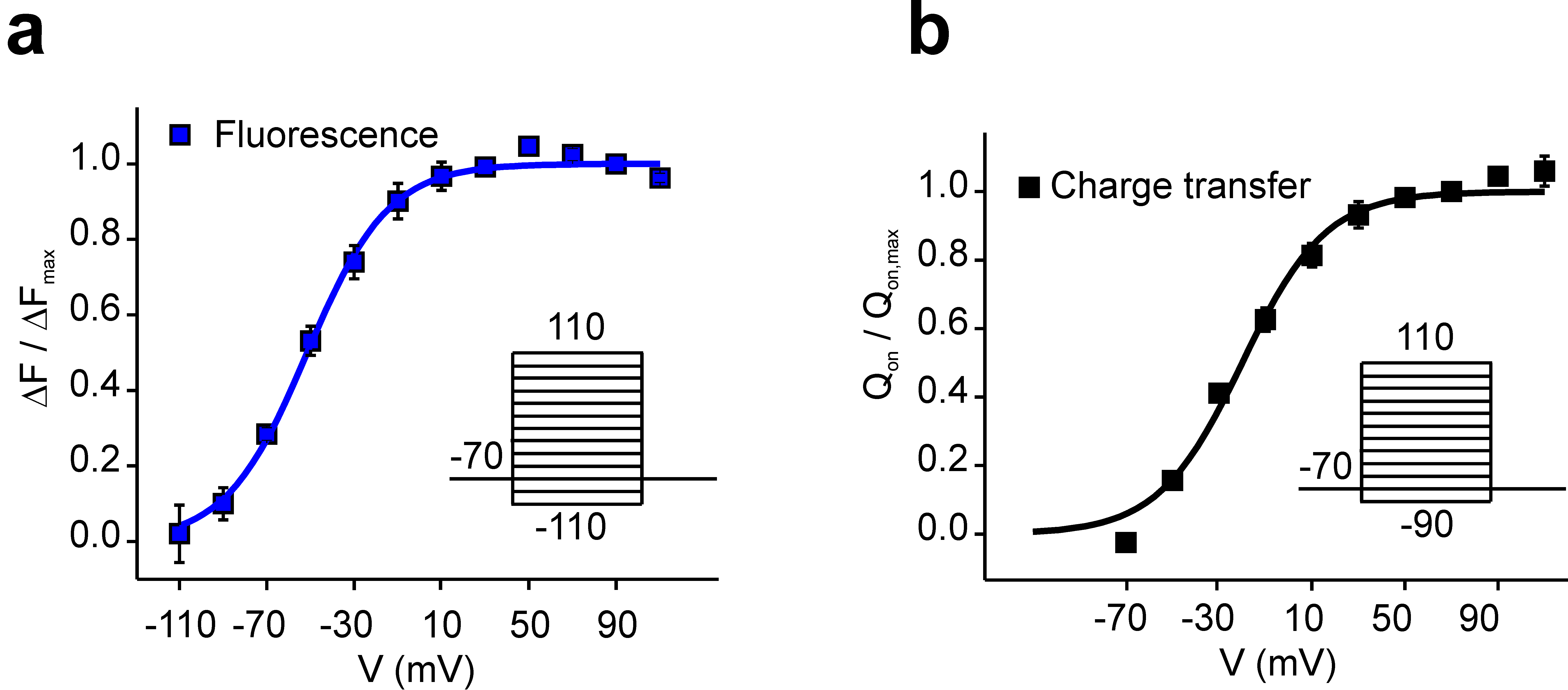

Supplement: Figure S3 — Voltage-dependency of VSFP3.1 fluorescence response and on-‘gating’ currents. (a) F-V relation for VSFP3.1 (n = 7). The change in cyan fluorescence elicited upon voltage steps between −110 mV and + 110 mV was normalized and fit to two-state Boltzmann distributions with mean values (V1/2 = −51.2±2.3 mV, a = 20.8±3.2). (b) Q-V relation for VSFP3.1 on-‘gating’ (n = 7). Currents were evoked by voltage steps between −70 mV and +110 mV, and the charge transfer was calculated and normalized. The data was fit to Boltzmann distributions with mean values (V1/2 = −18.3±1.5 mV, a = 19.1±1.2). (0.76 MB TIF) [file pone.0002514.s003.tif]
